# Outcome and risk of recurrence in a large cohort of idiopathic longitudinally extensive transverse myelitis without AQP4/MOG antibodies

**DOI:** 10.1186/s12974-020-01773-w

**Published:** 2020-04-23

**Authors:** Elisabeth Maillart, Françoise Durand-Dubief, Céline Louapre, Bertrand Audoin, Bertrand Bourre, Nathalie Derache, Jonathan Ciron, Nicolas Collongues, Jérome de Sèze, Mikael Cohen, Christine Lebrun-Frenay, Nawel Hadhoum, Hélène Zéphir, Romain Deschamps, Clarisse Carra-Dallière, Pierre Labauge, Philippe Kerschen, Alexis Montcuquet, Sandrine Wiertlewski, David Laplaud, Gwenaëlle Runavot, Sandra Vukusic, Caroline Papeix, Romain Marignier, on behalf of the OFSEP, SFSEP, and NOMADMUS study groups

**Affiliations:** 1 Department of Neurology; Centre de Référence des Maladies Inflammatoires Rares du Cerveau et de la Moelle, AP-HP, Hôpital Pitié-Salpêtrière, Paris, France; 2 Service de neurologie, sclérose en plaques, pathologies de la myéline et neuro-inflammation, and Centre de Référence des Maladies Inflammatoires Rares du Cerveau et de la Moelle, Hôpital Neurologique Pierre Wertheimer, Hospices Civils de Lyon, 69677 Lyon/Bron, France; 3 APHM, Hôpital de la Timone, Pôle de Neurosciences Cliniques, Service de Neurologie, Marseille, France; 4 Department of Neurology, University Hospital of Rouen, Rouen, France; 5 Department of Neurology, University Hospital of Caen, Caen, France; 6 Department of Neurology, University Hospital of Toulouse, Toulouse, France; 7 Department of Neurology, University Hospital of Strasbourg, Strasbourg, France; 8 Centre de Ressources et Compétence Sclerose en plaques (CRCSEP); Unité de Recherche Clinique Côte d’azur (UR2CA), CHU Pasteur 2, Nice, France; 9 Department of Neurology, University Hospital of Lille, Lille, France; 10 Department of Neurology, Fondation Ophtalmologique Adolphe de Rothschild, 25-29, rue Manin, 75940 Paris cedex 19, France; 11 Department of Neurology, University Hospital of Montpellier, Montpellier, France; 12 Department of Neurology, University Hospital of Luxembourg, Luxembourg, Luxembourg; 13 Department of Neurology, University Hospital of Limoges, Limoges, France; 14 Department of Neurology, University Hospital of Nantes, Nantes, France; 15 Department of Neurology, University Hospital of Saint-Pierre, Saint-Pierre, La Réunion France

**Keywords:** Longitudinally extensive transverse myelitis, Seronegative, Neuromyelitis optica, Outcome, Treatment

## Abstract

**Background:**

Longitudinally extensive transverse myelitis (LETM) is classically related to aquaporin (AQP4)-antibodies (Ab) neuromyelitis optica spectrum disorders (NMOSD) or more recently to myelin oligodendrocyte glycoprotein (MOG)-Ab associated disease. However, some patients remain negative for any diagnosis, despite a large work-up including AQP4-Ab and MOG-Ab. Data about natural history, disability outcome, and treatment are limited in this group of patients.

We aimed to (1) describe clinical, biological, and radiological features of double seronegative LETM patients; (2) assess the clinical course and identify prognostic factors; and (3) assess the risk of recurrence, according to maintenance immunosuppressive therapy.

**Methods:**

Retrospective evaluation of patients with a first episode of LETM, tested negative for AQP-Ab and MOG-Ab, from the French nationwide observatory study NOMADMUS.

**Results:**

Fifty-three patients (median age 38 years (range 16–80)) with double seronegative LETM were included. Median nadir EDSS at onset was 6.0 (1–8.5), associated to a median EDSS at last follow-up of 4.0 (0–8). Recurrence was observed in 24.5% of patients in the 18 following months, with a median time to first relapse of 5.7 months. The risk of recurrence was lower in the group of patients treated early with an immunosuppressive drug (2/22, 9%), in comparison with untreated patients (10/31, 32%).

**Conclusions:**

A first episode of a double seronegative LETM is associated to a severe outcome and a high rate of relapse in the following 18 months, suggesting that an early immunosuppressive treatment may be beneficial in that condition.

## Introduction

Longitudinally extensive transverse myelitis (LETM) is defined as a myelitis that extends over at least three vertebral segments in length at spinal cord MRI and is frequently associated to a severe clinical presentation [1]. Although LETM is classically related to AQP4-antibodies (Ab) neuromyelitis optica spectrum disorders (NMOSD) [2] or more recently to MOG-Ab associated disease [3], alternative diagnoses have to be excluded such as vascular, granulomatosis, paraneoplastic, metabolic, and infectious diseases [4]. However, despite a large work-up, some patients remain negative for any diagnosis, including AQP4-Ab and MOG-Ab associated disease. For these double seronegative LETM patients, current NMOSD criteria [5] are not fulfilled, and data about their natural history, disability outcome, and therapeutic recommendations are limited.

In this study, we aimed to (1) describe clinical, biological, and radiological features of double seronegative LETM patients; (2) assess the clinical course and identify prognostic factors; and (3) assess the risk of recurrence, according to maintenance immunosuppressive therapy.

## Methods

### Participants

Patients included in the study fulfilled the following criteria:

- First episode of myelitis, defined as progression to nadir symptoms as maximum duration between 4 h and 21 days following the onset of nadir, according the transverse myelitis Consortium Working Group [6]
- Associated with a T2 hyperintensity on spinal cord MRI extending over 3 vertebral segments
- Without previous neurological episode, especially optic neuritis
- Absence of both AQP4-Ab and MOG-Ab in serum detected either at onset of disease and during biological follow-up and tested using a cell-based assay in the French national reference laboratory [7]
- Age ≥ 16 years at onset of disease
- Brain MRI at admission not suggestive of multiple sclerosis (MS)
- Negative comprehensive work-up
- Minimum clinical follow-up required for at least 1 year

The initial work-up [1] included a first clinical research of extra-neurological symptoms. It consisted in standard biological blood analysis hematological, and biochemical assays, autoimmune assays (antinuclear antibodies, antiphospholipid antibodies, ANCA, protein electrophoresis), viral (Herpes-virus group, hepatitis B and C), bacteriological (syphilis, Lyme disease, tuberculosis) assessments, vitamin B12, full body CT scan, salivary gland biopsy, visual evoked potential, and ophthalmological examination. Brain and spinal cord MRI were performed according to the French National Guidelines (OFSEP) [8]. MRI centralized evaluations were performed by neuroradiologist experts, to rule out MS or other identified inflammatory diagnosis.

### Data collection

All requested information were retrospectively collected in a standardized evaluation form. Data collection was performed from January 2017 up to January 2019 and registered in the French nationwide database for NMOSD and associated neurologic disorders (NOMADMUS, a nested cohort of the Observatoire Français de la Sclérose en Plaques (OFSEP)). This form includes demographic data such as sex and age at disease onset; clinical data such as onset severity assessed by the Expanded Disability Status Scale (EDSS) score; biological data such as cerebrospinal fluid (CSF) pleïocytosis > 5 cells/mm^3^, protein level > 0.5 g/L, and presence of oligoclonal bands (OCB); radiological features with localization and extension of the LETM and description of the brain MRI; acute and maintenance therapy; time to the first relapse; duration of follow-up; and EDSS and radiological evolution during the follow-up. Clinical and radiological outcomes were assessed by EDSS and brain and spinal cord MRI at 6, 12, 18, and 24 months, when available, and at the last visit. AQP4-Ab and MOG-Ab in serum were performed at onset of disease and during biological follow-up and tested using a cell-based assay in the French national reference laboratory.

Double entry of the same patient was excluded by a single code of pseudonymization for each patient in the national database and stringent data management.

### Standard protocol approvals, registrations, and patient consents

All patients gave written informed consent to be included in NOMADMUS. Therefore, no additional consent or institutional review board approval was sought. All pseudonymized data were gathered in Pitié-Salpêtrière tertiary care center in Paris.

### Data availability

This study was done within the framework of OFSEP. Because of national confidentiality requirements, only anonymized data, not pseudonymized data, can be shared. While anonymization techniques might result in impoverishment of data (Article 29 of Directive 95/46/EC, Opinion 05/2014 on Anonymisation Techniques—0829/14/EN WP 216), data used for this study were only pseudonymized. However, access to OFSEP data to conduct a scientific project is possible by following the OFSEP data access process (ofsep.org/en/data-access) and with respect to French law.

### Statistical analyses

Statistical analysis was performed using the R software (version 3.4.0). Clinical characteristics were compared between patients with or without a relapse in the first 18 months of follow-up using Mann-Whitney *U* test or *χ*^2^test for gender repartition and presence of OCB. The independent predictive value of demographic and clinical characteristics to determine the risk of relapse within the first 18 months (milestone reached by every patient) was assessed with stepwise multilinear regression by using the Akaike Information Criterion (stepAIC function in R statistical package).

## Results

### Cohort description (Table 1)

Fifty-three patients fulfilled inclusion criteria: 28 women/25 men. Mean age at onset was 38 years (range 16–80). Median EDSS at nadir was 6 (range 1–8.5). Interval between onset of symptoms and spinal cord MRI was 19 days (range 1–117). Spinal cord lesions were predominantly localized in the thoracic (81%) and cervical (53%) areas with a mean lesion length of 6.2 (range 3–16) vertebral segments. Lesions were described as transverse in 45 patients (85%). No leptomeningeal enhancement was described.

**Table 1:** Clinical, radiological, and CSF data at first episode of LETM

Demographic data	Whole cohort	Non-relapsing patients in the first 18 months	Relapsing patients in the first 18 months
*n*	53	41	12
Female, *n* (%)	28 (52.8)	22 (54%)	6 (50%)
Age at onset, years median (range)	38 (16.5–80)	38 (16.5–80)	43.2 (26–65)
Follow-up, years median (range)	3.94 (1.5–12)	2.88 (1.5–11.3)	5.98 (3.91–12)
EDSS at nadir, median (range)	6 (1–8.5)	6.5 (1–8.5)	5.25 (1–8.5)
Spinal cord MRI data^a^			
· Cervical only	7	6	1
· Thoracic only	18	14	4
· Lumbar only	3	3	0
· Cervical and thoracic	14	10	4
· Cervical, thoracic and lumbar	7	6	1
· Thoracic and lumbar	4	2	2
· Cervical involvement, *n* (%)	28 (53%)	22 (54%)	6 (50%)
· Thoracic involvement, *n* (%)	43 (81%)	32 (78%)	11 (92%)
· Lumbar involvement, *n* (%)	14 (26%)	11 (27%)	3 (25%)
Laboratory data^b^			
CSF OCB, *n* = 50, *n* (%)	15 (30%)	12/38 (32%)	3/12 (25%)
CSF pleocytosis (> 5 cells/mm^3^), *n* = 52, *n* (%)	28 (54%)	22/40 (55%)	6/12 (50%)
Proteinorachy (> 0.5 g/L), *n* = 51, *n* (%)	24 (47%)	18/39 (46%)	6/12 (50%)

*LETM* longitudinal extensive transverse myelitis, *y* years, *m* months, *EDSS* Expanded Disability Status Scale, *CSF* cerebrospinal fluid, *OCB* oligoclonal bands

^a^Spinal cord MRI performed in the first month after symptoms onset

^b^Lumbar puncture performed during the first episode of LETM

Patients had normal brain MRI in 60% of cases, and not suggestive of MS in 40% of cases, with unspecific hyperintensities. No brain parenchymal or leptomeningeal gadolinium enhancement was identified.

Cerebrospinal fluid analyses at the acute phase revealed pleïocytosis (> 5 cells/mm^3^) in 56% of patients with a median cell count of 12/mm^3^ (range 0–990). Forty-seven percent of patients had an elevated protein level with a mean at 0.70 g/L (range 0.1–3.23 g/L), and oligoclonal bands were present in the CSF of 30% of patients.

AQP4-Ab and MOG-Ab were negative at least twice, at the onset and during the patients’ follow-up. For relapsing patients, AQP4-Ab and MOG-Ab were both re-assessed at the acute phase and remain negative.

For all the patients included, the complete work-up was negative, thereby did not reveal any underlying condition, especially other autoimmune disease.

Patients were treated in a median delay of 10 days (range 2–360) after the first symptoms, in 52 patients (98 %) by intravenous methylprednisolone (IVMP) (Table 2). A second line of treatment was performed in 24 patients (45%), including plasmapheresis for 17/24 patients (71%), IVMP for 5/24 patients (21%), or intravenous immunoglobulins for 2/24 patients (8%). The median delay for this second line of treatment was 16 days (range 6–146). In addition, oral corticosteroids were prescribed for 23 patients (43%), for a mean duration of treatment of 7.25 months (range 0.2–12 months).

**Table 2:** LETM initial treatment

	This cohort
Delay of treatment after first symptoms (mean, days)	10
First line of treatment (*n* = 53/53)	
Infusions of methylprednisolone	52 (98%)
Second line of treatment (*n* = 24/53)	
Plasmapheresis	17 (71%)
Infusions of methylprednisolone	5 (21%)
IV immunoglobulins	2 (8%)
Third line of treatment (n = 4/53)	
Infusions of methylprednisolone	2 (50%)
Plasmapheresis	2 (50%)
Relay by oral steroids (*n* = 23/53)	
Duration (data for 15 patients) months	7.2 (0.2–12)

*LETM* longitudinal extensive transverse myelitis, *IV* intravenous

### Clinical and radiological follow-up (Fig. 1)

Median EDSS improved from 6 to 5.25 at 6 months (range 0–8) (*n* = 50), 4.75 at 12 months (range 0–8) (*n* = 44), and 4.5 at 24 months (range 1–8) (*n* = 40). At the last follow-up for a median duration of 3.94 years (1.5–12), median EDSS remained 4 (range 0–8) (*n* = 53).

**Fig. 1:**
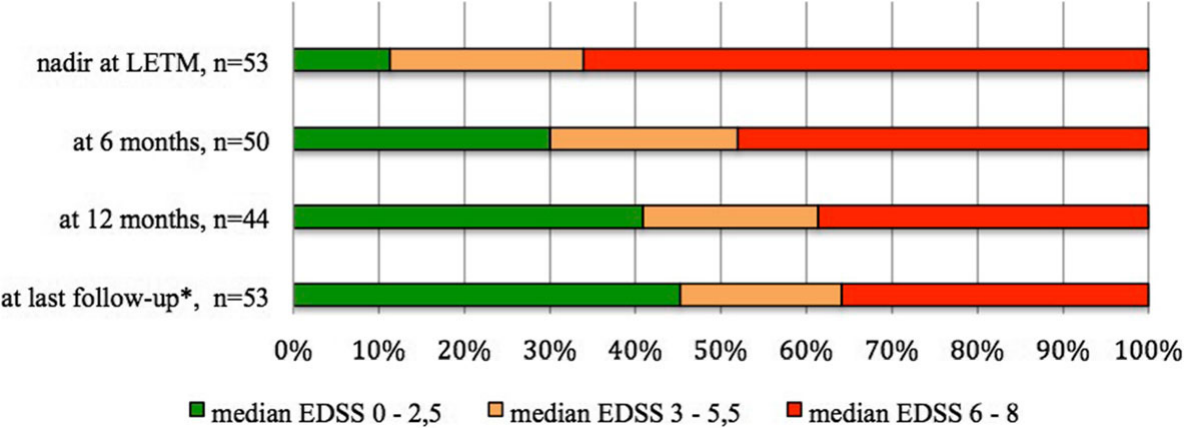
Clinical outcome of patients during the follow-up: evolution of EDSS over time. LETM, longitudinal extensive transverse myelitis; EDSS, Expanded Disability Status Scale

Twelve patients (24.5%) experienced at least another relapse (myelitis or optic neuritis) in the first 18 months, with a median interval between the 2 clinical events of 171 days (5.7 months). Among them, 6 fulfilled the 2015 NMOSD criteria. When comparing patients with a relapse in the first 18 months (*n* = 12) to patients without relapse (*n* = 41), we found no statistically significant difference for demographics and clinical variables (sex, age at onset, EDSS at nadir), biological (cellularity, presence of OCB), or radiological (location or number of vertebral segments) initial feature (Table 1). Because of the heterogeneity in the follow-up duration and the maintenance therapies, we focused on the relapses during the 18 first months (minimal duration of the follow-up in our cohort).

Concerning radiological evolution, no asymptomatic silent progression was reported. During MRI follow-up, all the LETM lesions decreased with spinal T2 high signal regression and disappearance of gadolinium enhancement the first 6 months, leading frequently to a spinal atrophy at the level of the LETM. No asymptomatic lesion appeared at brain MRI follow-up.

At the end of the follow-up, the final diagnosis was monofocal LETM in 35 patients, relapsing myelitis in 12 patients, and seronegative NMOSD according to 2015 criteria in 6 patients. No other disease such as sarcoidosis, neoplastic or paraneoplastic disorder, spinal cord infarction, or dural fistula was diagnosed at the end of the follow-up.

### Evaluation of maintenance therapies

Among the 29 patients receiving maintenance therapies, five patients had several successive maintenance treatments (3 patients with 2 different treatments and 2 patients with 3 different treatments). The main used drugs were: mycophenolate mofetil (16) and rituximab (9). Other immunosuppressants included: azathioprine (5), cyclophosphamide (3), mitoxantrone (1), methotrexate (1), and infliximab (1).

**Fig. 2:**
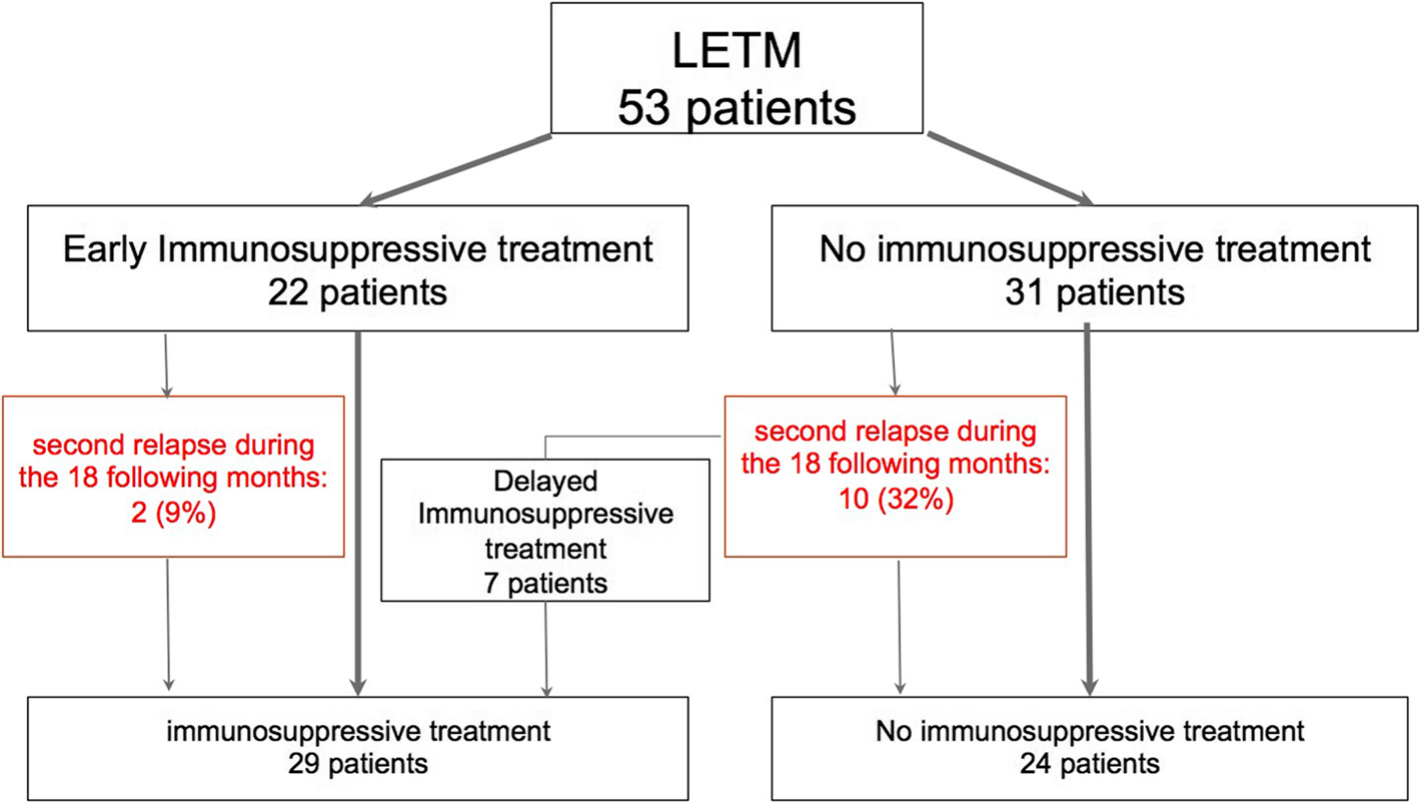
Flow-chart of the therapeutic strategy. LETM, longitudinal extensive transverse myelitis

Concerning the maintenance therapy, we defined 2 groups of patients: the first group received an early immunosuppressive treatment introduced in 6 months after the onset of the LETM management by neurologists. We identified 22 early immunosuppressive treated patients (Fig. 2**)**. The mean delay of therapy onset was 2.8 months (range 0.5–6.3). Among them, 2 patients (9%) had a relapse in the 18 following months (for both patients, 3 months after the LETM). The second group gathered the 31 untreated patients at the onset: among them, 10 patients (32%) experienced a second relapse in the 18 following months.

The difference in terms of risk of recurrence in the 18 following months between the early treated group and the untreated group was not statistically significant (*p* = 0.1 by *χ*^2^test). We identified 7 delayed immunosuppressive treated patients as they started their treatment after the second clinical event. None among them experienced a third relapse during the observatory period. We investigated the independent predictive value to determine the risk of at least one relapse during the first 18 months among the following dependent variables: sex, age at LETM, EDSS at the nadir, EDSS at M6, and start of early immunosuppressive treatment in the acute phase. The model enhanced that the unique predictive variable of the absence of a second clinical event during the first 18 months was the early treatment after the first episode of LETM (*p* = 0.048, *β*-estimate = − 0.23, *R*^2^ = 0.07; Odds Ratio 0.79 (95% confidence interval 0.634–0.993)).

## Discussion

This work provides the largest cohort to date of 53 patients with AQP4-Ab and MOG-Ab negative LETM, from the French, multicentric, nationwide observatory NOMADMUS, a nested cohort of the OFSEP. All these patients underwent a large standardized and comprehensive work-up to rule out conventional differential diagnosis such as vascular (i.e., dural fistula), granulomatosis, paraneoplastic, metabolic, and infectious diseases. Clinical and radiological data at onset and outcome have been accurately collected with evaluation of treatment effect. In this cohort, the majority of patients experienced a severe LETM (median nadir EDSS of 6.0). The outcome was poor with very partial recovery for most of the patients (median EDSS at 4), with a median follow-up of nearly 4 years. Besides, recurrence was observed in 24.5% of patients, mainly in the first year. The risk of recurrence trended to be higher in the group of patients with delayed immunosuppressive treatment (32%) than in the group of patients with early immunosuppressive treatment (9%).

Before starting a longer term immunosuppression, neurologists must be very stringent, trying to exclude difficult differential diagnosis [9] such as neuro-sarcoidosis, spinal cord infarction [10], or dural fistula. Neurologists need to be aware of evocative MRI patterns of alternative diseases: leptomeningeal enhancement or trident pattern of enhancement in neurosarcoidosis, compressive aspect or mass lesion in a spinal tumor, patchy anterior horn T2 hyperintensities in a spinal cord infarction, and serpiginous vessels in a spinal dural arteriovenous fistula, as proposed in series of idiopathic transverse myelitis [9]. In this study, to avoid misdiagnosis, inclusion criteria were stringent, and MRI was performed following the OFSEP protocol, centrally analyzed by neuroradiological experts in the field, which is a guarantee of quality. Beyond MRI pattern, the question of the exhaustive work-up is crucial is AQP4/MOG seronegative LETM. As the reported cases are retrospective and collected data are multicentric, some explorations were realized case by case. For example, PET scan is known to be more sensitive that CT scan to detect systemic sarcoidosis, for the staging and identification of occult sites and sites suitable for biopsy [11]. But for historical and availability issues, PET scan was not performed in every patient. Nowadays, realization of PET scan needs to be discussed in the work-up of an unexplained LETM. Moreover, infectious etiologies need to be rule out with comprehensive CSF analysis, including rare etiologies such as enterovirus D68.

Available data on LETM with negative AQP4-Ab and MOG-Ab are scarce (Table 3). In a Korean cohort [12] including 42 patients with double seronegative LETM in comparison to our findings, the outcome was better (median EDSS score at 2.5), but the attack was also initially less severe at nadir (median nadir EDSS at 3.0). The rate of relapse was higher (71%) with a median time to the first relapse of 11.5 months (range 2–72). However, 57% of patients were untreated at the final follow-up, versus 45% in our study. Two Spanish cohorts [13, 14] found similar results, with high EDSS at nadir, and a slightly higher relapse rate (28.6–30%) but with better outcome (median EDSS 2.5 at the end of follow-up). However, inclusion criteria differed, with MOG-Ab status unknown on one hand [13], and inclusion of patients with LETM related to MS on the other hand [14]. An English cohort [15] including patients with MS or ADEM found similar high EDSS at nadir associated to a better recovery (EDSS at 3). The relapse rate was increased to 31%, maybe because of inclusion of MS patients. Finally, a monocenter retrospective study including 192 patients with transverse myelitis (and not necessary LETM) found a rate of recurrence around 57% [16]. Multiple independent risk factors for recurrence were found, such as African American race, female sex, and LETM at onset, maybe partly driven by a greater likelihood of developing NMOSD. Overall, we need to keep in mind that the effect and the early initiation of the attack treatment are important factors when assessing clinical recovery, as it has been previously demonstrated for IVMP or PLEX [17, 18].

**Table 3:** Comparison of different cohorts of seronegative LETM

Publication	Patients number	Age at LETM onset (range) years	Sex ratio	AQP4 Ab	MOG Ab	Median EDSS at nadir (range)	Chronic treatment	At least one relapse after	Follow-up duration, (range)	Median EDSS at follow-up (range)
**Sepulveda et al.** [13]	23	Median 44.5 (20–77)	16 F 7 M	2	NA	7 (3–9)	12/23, 52%	6/20, 30%	Median 32 months (6–54)	2.5 (0–8)
**Kitley et al.** [15]	32 including MS, ADEM	Mean 37.74 (± 16.07)	14 F 18 M	0	6	8 (3–8)	NA	31%	Median 25 months (1.9–169.4)	EDSS at recovery 3 (3–8)
**Huyn et al.** [12]	42	Mean 43.1 (± 9.8)	9 F 33 M	0	0	3 (3–8.5)	NA	30, 71%	Mean 5.4 ± 2.6 years	2.5 (1–6)
**Cobo-Calvo et al.** [14]	56 including MS	Median 39.9 (32.3–58.1)	30 F 16 M	0	13	5.0 (3.5–7.8)	19/56, 33.9%	16, 28.6%	Median 42.2 months (25–79.5)	2.5 (1.5–4.8)
**This cohort**	53	Median 38 (16–80)	28 F 25 M	0	0	6 (1–8.5)	29/53, 55%	12/53, 24.5%	Median 3.94 years (1.5–12) mean 4.47 years	4 (0–8)

*Ab* antibody, *EDSS* Expanded Disability Status Scale, *F* female, *M* male, *MS* multiple sclerosis, *ADEM* acute disseminated encephalomyelitis

Beyond the clinical phenotype of LETM, the severity of double seronegative NMOSD has been previously pointed. In a cohort of 181 patients fulfilling the 2006 NMO criteria or NMO limited forms with AQP4-Ab [19], patients with AQP4-Ab or double seronegative had significant poorer outcome than patients with 2006 NMO criteria and MOG-Ab. Even if some studies report functional and microstructural damages in MOG-Ab optic neuritis [20, 21], visual recovery measured by visual acuity seems poorer in double seronegative and AQP4-Ab patients with optic neuritis [22]. Concerning radiological features, LETM lesions in patients fulfilling 2006 NMO criteria differentiated with fragmentation and atrophy [23]. In the Spanish cohort of double seronegative LETM, lesions decreased, disappeared, or remain stable: atrophy was reported in 14% of patients. On the opposite of MOG-associated disease, similar clinical evolution in patients with seronegative NMOSD and AQP4-Ab suggest a common physiopathology with acute axonal injury, poor recovery, and spinal atrophy.

Indication of an immunosuppressive therapy after a double seronegative LETM has not been studied in a randomized prospective trial because of the scarcity of this disorder. In this cohort, the difference in terms of risk of recurrence relapse in the first 18 months between the patients treated after the LETM or untreated patients was not statistically significant, probably due to the small number of patients in our cohort. However, the only predictive variable of the absence of a second clinical event during the first 18 months was the early immunosuppressive treatment after LETM suggesting that early treatment might decrease the risk of recurrence. Mycophenolate mofetil and rituximab have been preferentially used because of probable efficacy in NMOSD, especially with AQP4-Ab [24–26]. More data are needed, in the light of the recently described lower efficacy of anti-CD20 in the MOG-antibody associated diseases [27]. Finally, as immunosuppressive treatments take time to be effective, an association with oral steroid should be prescribed during the first 6 months to prevent early relapses and to avoid changing too early the maintenance therapy [24].

## Limitations

First, some limitations of this study are inherent due to its retrospective design. Then, even we used strict inclusion criteria, this cohort is possibly heterogeneous, which is in part due to multicenter effect. Despite this heterogeneity, the strict inclusion criteria, the common negative initial work-up, the expertise of the different centers participating to this work, and the long clinical and MRI follow-up allow speculating that our patients share common features.

Second, even if a clinical neuro-ophthalmological evaluation and visual evoked potentials (VEP) were realized, an exhaustive visual system assessment was not systemically performed, especially with optical coherence tomography (OCT). Indeed, alteration of VEP and/or OCT is not considered in the 2015 NMOSD criteria. However, in some NMOSD patients without optic neuritis history, infraclinical foveal thickness in OCT [28] or slight increasing of latency of P100 in VEP [29] was reported. Association of VEP and OCT could be interesting prognostic biomarkers in double seronegative LETM.

Third, management of maintenance therapy varied according to the centers and the local practices, in particular with the increasing use of rituximab after LETM in suspected NMOSD during these last 10 years.

Fourth, GFAP antibodies were not tested retrospectively in the CSF of the patients in our cohort. This recent entity is defined as a distinct autoimmune astrocytopathy, responsible for a corticosteroid-responsive meningo-encephalomyelitis. Nevertheless, in the recent publication of 13 patients with LETM associated to GFAP-antibodies [30], all have extra-spinal symptoms simultaneously or preceding myelitis onset, on the contrary to our patients. Such as recent identification of GFAP antibodies, other antibodies could be identified in the future and help to break down this heterogeneous entity of seronegative LETM.

## Conclusion

In our cohort, the outcome of the 53 patients with double seronegative longitudinal extensive transverse myelitis was frequently severe, and the rate of relapse in the following 18 months was high. After a comprehensive work-up and an attentive analysis of both brain and spinal cord MRI to rule out any possible alternative diagnoses, an early immunosuppressive treatment may be beneficial in that condition. Better knowledge of physiopathology and identification of risk factors for recurrence are required, yielding to prospective randomized controlled trials.

## Data Availability

This study was done within the framework of OFSEP. Because of national confidentiality requirements, only anonymized data, not pseudonymized data, can be shared. While anonymization techniques might result in the impoverishment of data (Article 29 of Directive 95/46/EC, Opinion 05/2014 on Anonymisation Techniques—0829/14/EN WP 216), data used for this study were only pseudonymized.

## Abbreviations

LETM: Longitudinal extensive transverse myelitis
EDSS: Expanded Disability Status Scale
CSF: Cerebrospinal fluid
OCB: Oligoclonal bands
Ab: Antibody
MS: Multiple sclerosis
ADEM: Acute disseminated encephalomyelitis
